# Green bodies: Inclusions in a critically ill COVID‐19 patient

**DOI:** 10.1002/jha2.197

**Published:** 2021-05-04

**Authors:** Ioulia Chaliori, Nikolaos J. Tsagarakis, Sofia Chaniotaki, Georgios Androutsos, Dimitrios Liakopoulos, Elpiniki Kritikou‐Griva

**Affiliations:** ^1^ Hematology Laboratory General Hospital of Athens “G. Gennimatas,” Athens Greece

 
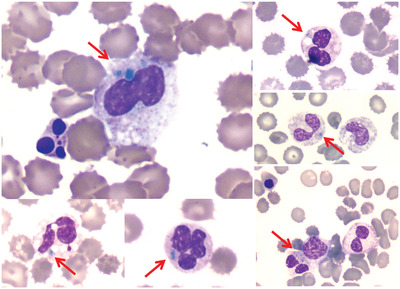


A 55‐year‐old male with comorbidities was diagnosed positive to a highly transmissible SARS‐COV‐2 variant (with the D614G substitution) and admitted to our hospital with moderate COVID‐19. His respiratory function progressively worsened and he was admitted to the ICU (12 days later) under mechanical ventilation. Green‐blue inclusions were observed in a few neutrophils at his blood smear a few days later, as well as vacuolated neutrophils, dysplasia of granulocytes, apoptotic cells, echinocytes, and nucleated red blood cells. At that time the patient was hemodynamically unstable, oliguric, and with signs of metabolic acidosis. He had elevated LDH (56.94 ukat/L) and ferritin (33511 μg/L), moderately increased AST (4.48 ukat/L) and ALT (1.74 ukat/L), thrombocytopenia (10^9^/L), and increased d‐dimers (28.6 mg/L) and lactate (4.5 mmol/L). The patient died 6 days later.

“Green bodies” are rare cytoplasmic inclusions of high lipid content in neutrophils and monocytes of peripheral blood, which have been observed in critically ill patients suffering from liver necrosis, severe tissue damage, lactic acidosis, and more recently from COVID‐19. Usually underreported, they seem to be an ominous prognostic sign, as in this case, and their early detection may be of clinical importance.

